# The cost-effectiveness of smoking cessation support delivered by mobile phone text messaging: Txt2stop

**DOI:** 10.1007/s10198-012-0424-5

**Published:** 2012-09-09

**Authors:** Carla Guerriero, John Cairns, Ian Roberts, Anthony Rodgers, Robyn Whittaker, Caroline Free

**Affiliations:** 1 Health Services Research and Policy, London School of Hygiene and Tropical Medicine, London, UK; 2 The George Institute for Global Health, University of Sydney, Sydney, Australia; 3 Clinical Trials Research Unit, University of Auckland, Auckland, New Zealand; 4 Population Health, London School of Hygiene and Tropical Medicine, London, UK

**Keywords:** Smoking cessation aid, Economic evaluation, Text message, I18

## Abstract

**Background:**

The txt2stop trial has shown that mobile-phone-based smoking cessation support doubles biochemically validated quitting at 6 months. This study examines the cost-effectiveness of smoking cessation support delivered by mobile phone text messaging.

**Methods:**

The lifetime incremental costs and benefits of adding text-based support to current practice are estimated from a UK NHS perspective using a Markov model. The cost-effectiveness was measured in terms of cost per quitter, cost per life year gained and cost per QALY gained. As in previous studies, smokers are assumed to face a higher risk of experiencing the following five diseases: lung cancer, stroke, myocardial infarction, chronic obstructive pulmonary disease, and coronary heart disease (i.e. the main fatal or disabling, but by no means the only, adverse effects of prolonged smoking). The treatment costs and health state values associated with these diseases were identified from the literature. The analysis was based on the age and gender distribution observed in the txt2stop trial. Effectiveness and cost parameters were varied in deterministic sensitivity analyses, and a probabilistic sensitivity analysis was also performed.

**Findings:**

The cost of text-based support per 1,000 enrolled smokers is £16,120, which, given an estimated 58 additional quitters at 6 months, equates to £278 per quitter. However, when the future NHS costs saved (as a result of reduced smoking) are included, text-based support would be cost saving. It is estimated that 18 LYs are gained per 1,000 smokers (0.3 LYs per quitter) receiving text-based support, and 29 QALYs are gained (0.5 QALYs per quitter). The deterministic sensitivity analysis indicated that changes in individual model parameters did not alter the conclusion that this is a cost-effective intervention. Similarly, the probabilistic sensitivity analysis indicated a >90 % chance that the intervention will be cost saving.

**Interpretation:**

This study shows that under a wide variety of conditions, personalised smoking cessation advice and support by mobile phone message is both beneficial for health and cost saving to a health system.

## Introduction

The txt2stop trial randomised 5,800 smokers aged 16 years or older to personalised smoking cessation advice and support by mobile phone message or to a control group [1]. Participants in the intervention arm received five text messages per day for the first 5 weeks and three per week for the next 26 weeks. Participants in the control group received texts every two weeks related to the importance of trial participation. Mobile phone text messaging smoking cessation support doubles (10.7 vs 4.9 %) biochemically verified smoking cessation at 6 months (relative risk 2.20, 95 % CI 1.80–2.68 *P* < 0.0001) compared to a control group using any existing smoking cessation support of their choice. The intervention is effective in all socio-economic groups, and in younger and older smokers [1]. A detailed description of the development of the txt2stop intervention is reported elsewhere [2, 3].

Interventions to encourage smoking cessation must be assessed not only for effectiveness but also in terms of value for money [4]. Existing smoking cessation interventions that have been shown to be cost effective (in a UK context a cost per QALY gained of <£20,000) include group counselling, one-to-one counselling, telephone counselling, and medications, such as nicotine replacement therapy and varenicline [5]. To our knowledge, there are no previous cost-effectiveness evaluations of smoking cessation interventions utilising mobile devices. This study assesses the cost-effectiveness of text-based support when added to the treatments used in the control arm of the txt2stop trial. At randomisation, 818 participants were using additional smoking cessation support: 82 % used medication (NRT, buproprion or varenicline), 4 % used a telephone helpline, 3 % used group or individual counseling, and 12 % used other support.

## Methods

This analysis applied a cohort simulation model to determine the cost-effectiveness of text-based support in addition to current practice versus current practice alone. Current practice is defined as the mix of interventions currently available in the UK to help people stop smoking (as represented in the txt2stop trial). One in seven of the trial participants were using additional smoking cessation support at randomisation (82 % used nicotine replacement therapy, buproprion or varenicline; 4 % a telephone helpline; 3 % group or individual counselling; and 12 % used other support).

### Model

**Fig. 1:**
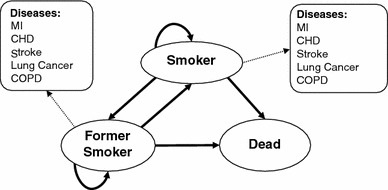
Markov model

This study uses a model adopted in previous economic evaluations of smoking cessation interventions conducted in UK from a health service perspective [5, 6]. The Markov state transition model (Fig. 1) used in the study by Flack et al. [5] is populated using the most recent UK data. At the start of the analysis, the simulated population consists entirely of smokers. A 6-month cycle is adopted, with transitions between smoking status occurring every 6 months according to the probability of remaining, in or moving to, one of three mutually exclusive states: smoker, former smoker, and dead. In each cycle it is assumed that both former and current smokers have a chance of developing the five main health consequences of smoking: lung cancer, stroke, myocardial infarction (MI), chronic obstructive pulmonary disease (COPD), and coronary heart disease (CHD) [7]. A lifetime horizon is chosen in order to calculate the incremental cost of text-based support, and the life years (LY) and quality adjusted life years (QALY) gained. This time period is necessary to allow for the inclusion of all costs and effects of the intervention. All costs are expressed in UK pounds (£) in terms of financial year 2009–2010. Costs are estimated from an NHS perspective and the discount rate used is 3.5 % for cost and outcomes as per NICE guidance [8]. In order to allow comparison with previous economic evaluations of smoking cessation interventions in UK we have used the same data sources as Flack et al. [5]; more recent data being used where available.

### Study population

The number of LYs and QALYs gained by text-based support, and thus its cost-effectiveness, will depend on the initial age of the smokers as smoking mainly causes health conditions that need a long time period to develop after exposure to smoking. To allow for this, the analysis is conducted separately for three age groups: <30 years (30 % of the trial population), 30–40 years (31 %), and >40 years (39 %). The mean age for each age group was 24, 35 and 48 years, respectively, based on the txt2stop trial data. These values are adopted as the starting age in the cohort models. The number of LYs and QALYs gained also depends on the gender of the smokers. It is well established that women live longer than men but with a higher burden of disabilities [9]. In order to account for this, the analysis is conducted for each age group separately for men and for women. The overall results (number of life years, QALY and disease costs) are calculated as a weighted average of the results for the three age groups using the gender proportions observed in the trial (male 55 %, female 45 %).

### Probabilities

The relative risk of quitting at 6 months for text-based support observed in the trial, 2.20 (95 % CI 1.80–2.68, *P* < 0.0001), is applied to the quit rate at 6 months in the control group (4.9 %) [1]. A 21 % relapse rate is assumed between 6 and 12 months for both the control and the treatment group [10]. A recent meta-analysis of 12 trials estimates that there is no difference between control and treatment groups in the relapse to smoking after 1 year of cessation (OR 1.11, 95 % CI 0.78–1.59). Based on this latter study, a lifetime relapse rate of 30 % among those who have quit for 12 months is assumed in the model [11].

Failure to take account of the background quit rate would lead to an overestimate of the effectiveness of text-based support, since a number of the additional quitters would have quit anyway in the future in the absence of test-based support. The background quit rate is likely to vary across age groups. The markedly higher proportion of ex-smokers among those aged 55 and over might indicate a higher background quit rate in this age group [12]. Also the background quit rate may be increasing as more support for smoking cessation becomes available. However, in the absence of good data, and following previous studies, a background quit rate of 2 % per year is assumed for all smokers (with or without text-based support) for all 6-month simulation cycles independently of the age and gender of the smokers [13].

Mortality rate in the general population by age and gender were retrieved from the Health Survey for England [12]. The prevalence of smokers, never smokers and quitters by age and gender in the UK population was obtained from the 2009 Office of National Statistics household survey [14]. The relative risk of dying of smokers versus never smokers and quitters by age was retrieved from a study conducted by Doll et al. [15]. These data were combined to calculate the probability of dying for a single individual in the cohort changes within each cycle according to the individual age, gender and smoking status (former smokers, smokers). As for previous studies the formula used was the following:

$$ \begin{aligned} {\text{Mortality rate}}_{ag} = & \, \left( {{\text{Mortality smoker}}_{a} *{\text{Prevalence of smoker}}_{ag} } \right) \, \\ & + \, \left( {{\text{Mortality former smoker}}_{a} *{\text{Prevalence of former smoker}}_{ag} } \right) \, \\ & + \, \left( {{\text{Mortality of never smoker}}_{a} *{\text{Prevalence of never smoker}}_{ag} } \right) \\ \end{aligned} $$

Where *a* is the age group and *g* is gender. The estimated mortality rates used to populate the model are reported in Table 3 of the “Appendix”.

Similarly, the probability of experiencing smoking-related diseases is estimated for each gender and age separately using the formula reported below (See Table 4 “Appendix”) [5]:

$$ \begin{aligned} {\text{Disease prevalence}}_{ag} &= \, \left( {{\text{Disease prevalence smoker}}_{ag} *{\text{ Prevalence of smoker}}_{ag} } \right) \, \\ & + \, \left( {{\text{Disease prevalence of former smoker}}_{ag} *{\text{prevalence of former smoker}}_{ag} } \right) \, \\ & + \, \left( {{\text{Disease prevalence of never smoker}}_{ag} *{\text{Prevalence of never smoker}}_{ag} } \right) \\ \end{aligned} $$

As with previous studies we include overall mortality by smoking status and did not consider disease-specific mortality in order to avoid double counting. Diseases within each cycle were assumed to be mutually exclusive (within each 6 months individuals can experience only one of the five diseases, survive with no disease or die). This assumption is consistent with previous studies.

As in Flack et al. [5] and Raikou and McGuire [6], the prevalence rates for lung cancer and COPD are taken from Forman et al. [16] and Britton [17], respectively (See “Appendix”). Prevalence of CHD, MI and stroke are taken from the study by Allender et al. [18] (See “Appendix”). The probability of developing lung cancer by smoking status and gender comes from Peto et al. [19]. while the relative risks of the other smoking-related diseases (CHD, MI, COPD and stroke) are from a study on the health consequences of smoking conducted by the Department of Health and Human Services [7] (See “Appendix”).

### Health state values

The health state values assigned to smoking-related diseases and, in absence of these diseases, to smoking status follow Flack et al. [5]. Diseases such as lung cancer, COPD and stroke present several phases of disease progression. For example, Tengs and Wallace [20] identify four health state values according to the type of stroke: minor stroke, moderate stroke, acute stroke requiring hospitalization and major stroke. Similarly, health state values associated with lung cancer are affected by the type of treatment undertaken and the stage of the disease. However, to assign different values according to the severity level of the disease requires knowledge of the proportion of smokers and previous smokers in each of these states. Lacking these data, simple averages of the available values for each of the diseases are used as in previous evaluations [5]. The values used for each disease are: 0.58 for lung cancer, 0.48 for stroke, 0.80 for CHD and MI (the estimate for MI is an average of the values reported by Tengs and Wallace [20] for MI of different disease severities), and 0.73 for COPD (an average of the different values for COPD severity estimated by Rutten-van Molken et al. [21]). Finally, different values are assigned to smokers (0.75) and former smokers (0.78) as reported in the UK study conducted by Tillman and Silcock [22].

### Costs

The cost of text-based support per smoker is the sum of three elements: the cost of enrolling smokers (including the cost of collecting information about age, gender education etc.), the cost of text messages (including the cost of setting a short code), and any royalty paid for use of the intervention.

Smokers wishing to use text-based support can register directly online or by SMS. The cost of web site maintenance is assumed to be zero in this analysis because the same site is used for other types of smoking cessation services. The cost of text messages per smoker, £16.12, includes the cost of setting up a short code (£0.06/participant), and the cost of sending the messages (£14.51). The lack of data on the proportion of smokers and former smokers at each disease stage does not allow consideration of how costs vary according to the severity of these diseases. Average cost estimates were used in the absence of these data. For example, in the case of stroke the estimated annual total cost of stroke in UK was divided by the number of people who experienced the disease [5]. The annual costs assigned to each of the smoking-related diseases are lung cancer (£5,921), stroke (£2,218), MI (£2,341), COPD (£997), and CHD (£1,144) [23–27]. All the costs are inflated to 2009–2010 prices using the hospital and community health services price index.

### Sensitivity analysis

Deterministic sensitivity analyses and a probabilistic sensitivity analysis (PSA) were performed to assess parameter uncertainty. The impact of variations in the effectiveness of text-based support on cost-effectiveness was investigated by assuming that the relative risk ranged between 1.80 and 2.68 (the 95 % confidence interval around the effect observed in the txt2stop trial). Further analyses were performed to estimate the cost-effectiveness of text-based support for different lifetime relapse rates, 21 %, used as the lower value (reported by McGhan and Smith [28]) and 50 % (the highest value reported in the literature for the relapse rate between 6 and 12 months [29]).

The lower value for the background smoking cessation rate in the one-way sensitivity analysis (1.2 %) is the historic rate over the past 40 years in England, while the upper value (2.8 %) is the highest background cessation rate suggested by West [13]. In the base case analysis, advertising cost is assumed to be zero and it is assumed that 100 % of smokers will register online. It is not known whether the NHS would advertise the intervention using pre-existing channels at relatively low marginal cost or whether advertising on the radio/internet/TV will be utilised. In order to account for this element of uncertainty, the incremental cost-effectiveness of text-based support is estimated assuming an intervention cost ranging from £15 per smoker (assuming that all the smokers register on-line, no crave messages and 10,000 users per short code) to £60.22 (assuming an additional advertising cost of £44, as observed in the txt2stop trial). These figures are used for illustrative purposes. Given large-scale implementation of text-based support, advertising costs are likely to be lower than those incurred when advertising the opportunity to participate in a smoking cessation trial. There are some additional costs which could arise in practice, such as royalty payments for the use of the IT program for the text-based support intervention, and management costs to co-ordinate the provision of the service. Both of these would be influenced strongly by the scale of text-based support were it to be implemented, the larger the scale the lower the cost per smoker. To investigate the impact of these potential additional costs, the analysis was re-run with additional costs of £1 and £35 per smoker enrolled.

A second order Monte Carlo simulation with 1,000 iterations was used to assess the influence of parameter uncertainty on the study results. Parameters were considered independent. Following suggested practice, a lognormal distribution was assigned to relative risks, and beta distributions were assigned to the lifetime relapse rate, baseline quit rate and health state values [30] (See “Appendix”). A gamma distribution was adopted for unit costs. Each variable estimate was derived from its probability distribution (See “Appendix”).

Cost-effectiveness acceptability curves (CEACs) were constructed to represent uncertainty regarding the parameters of the model. The net monetary benefit from text-based support was estimated for each simulation using the following equation:

$$ {\text{Net monetary benefit}} = \lambda *(E_{T2S} - E_{CP} ) - ({\text{COST}}_{T2S} - {\text{COST}}_{CP} ) $$

where: λ represents the “willingness to pay” per QALY gained, (*E*_T2S_ − *E*_CP_) is the incremental effectiveness (number of QALY gained) of text-based support, and (COST_T2S_ − COST_CP_) is the incremental cost of text-based support. CEACs plot the proportion of simulations for which text-based support is cost-effective for a willingness to pay per QALY from £0 to £4,000.

## Results

The cost of text-based support per 1,000 enrolled smokers is £16,120, which, given an estimated 58 additional quitters at 6 months, equates to £278 per quitter. However, once the avoided future NHS costs (as a result of reduced smoking) are taken into account, text-based support would be cost saving as observed in Table 1 for all three age groups. It is estimated that 18 LYs are gained per 1,000 smokers receiving text-based support and 29 QALYs are gained (of which 27 are attributable to a reduction in smoking-related diseases).

**Table 1:** Incremental costs, life years (LYs) gained and quality-adjusted life years (QALYs) gained per 1,000 enrollees

	25 year-old^a^	35 year-old^a^	48 year-old^a^	Weighted average^b^
Cost without text-based support	£3,177,185	£4,690,512	£7,446,703	£5,299,712
Cost with text-based support	£3,166,119	£4,660,193	£7,374,176	£5,258,203
LYs without text-based support	23,546	21,591	18,244	20,859
LYs with text-based support	23,555	21,607	18,271	20,877
QALYs without text-based support	17,772	16,136	13,341	15,528
QALYs with text-based support	17,792	16,163	13,379	15,557
Incremental cost	−£11,066	−£30,320	−£74,214	−£41,509
Incremental LYs	9	16	27	18
Incremental QALYs	20	27	38	29

^a^These ages represent the <30 years, 30–40 years and >40 years groups in the simulation

^b^Weights are from the txt2stop trial

One-way sensitivity analyses are reported (for all smokers) in Table 2. If a much lower estimate of the treatment effect is assumed, other parameters remaining the same, text-based support continues to be cost saving while at the same time producing gains in LYs and QALYs. Varying the relapse rate and the baseline quit rate does not change the finding that text-based support is health improving and cost saving. If a higher intervention cost is assumed and advertising costs are similar to those observed in the txt2stop trial, then the incremental cost-effectiveness would be £141 per LY gained and £89 per QALY gained. If a management cost of £35 is charged per participant the intervention is still cost-saving assuming a health service perspective.

**Table 2:** One-way sensitivity analyses

	Incremental cost per LY	Incremental cost per QALY
Relative risk continuous abstinence at 6 months (1.80:2.68)	NA:NA^a^	NA:NA
Lifetime relapse rate (21:50 %)	NA:NA	NA:NA
Baseline quit rate (1.2:2.8 %)	NA:NA	NA:NA
Cost of intervention per smoker (£6.70:£62.30)	NA:£141	NA:£89
Royalty, management cost per smoker (£1:£35)	NA:NA	NA:NA

^a^Not applicable because health improving and cost saving

**Fig. 2:**
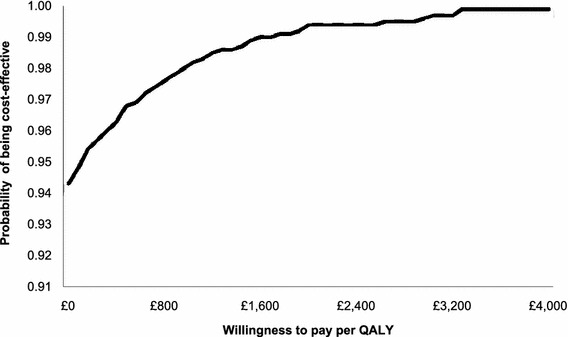
Cost-effectiveness acceptability curve

The PSA, in which distributions are assumed to reflect uncertainty about assumptions regarding the relative risk, the lifetime relapse rate, the baseline quit rate, and the unit costs, implies that there is a >90 % chance that the intervention will be cost saving (see Fig. 2).

## Discussion

This economic evaluation shows that text-based smoking cessation support (provided in addition to the existing range of smoking cessation services) produces health benefits and reduces health service costs. Moreover, sensitivity analyses indicate that text-based support remains cost effective under a wide range of alternative assumptions.

The primary strength of this evaluation of text-based support for smoking cessation is that it uses a valid and precise estimated treatment effect. A further strength is that the analysis is based on an established economic model. Nonetheless, a number of limitations must be considered when interpreting the study findings. As with previous economic evaluations this study potentially undervalues the benefits of text-based support as a smoking cessation intervention in that it does not take into account the effects of reduced passive smoking, nor does it account for short-term effects (e.g. reduction in respiratory problems) associated with smoking cessation [5, 6, 31, 32], or a wide range of other less common smoking-related diseases [33]. For this reason it also underestimates the potential savings from the intervention because it does not account for the cost (differences) of complication-free health states (smokers without complications are likely to cost more to the NHS rather than former smokers without complications). Another limitation of this study is that the costs and the health state values associated with different smoking-related diseases are averages across different severity levels. This is because of the lack of information on the proportion of smokers, quitters and never smokers experiencing the different severity levels.

As always, there is uncertainty regarding the values of the various model parameters. The likely cost of text-based support if it were to be implemented widely would depend, in part, on the numbers using the service, since this may influence the cost of text messages and royalty payments.

Finally, this study did not account for non-smoking-related medical costs. A recent study conducted in the Netherlands showed that, while smoking cessation interventions reduce the number of smokers and the medical cost of treating a range of medical conditions, such as stroke, cardiovascular diseases etc., associated with smoking, former smokers have higher lifetime medical costs because they survive longer [34, 35]. If a perspective were adopted where non-smoking-related medical costs were to be considered, the estimated savings associated with text-based support will have been over-estimated.

Care must be taken when comparing the cost per quitter, cost per LY gained and cost per QALY gained reported in this study with those from other studies because incremental cost-effectiveness will depend, in part, on the comparator. In this study the comparator was current practice, here defined as the mix of smoking cessation interventions accessed by those in the control arm of the txt2stop trial. The results of this study are consistent with previous economic evaluations that show that smoking cessation interventions are highly cost effective. The cost per quitter for text-based support appears lower than for some other smoking cessation interventions. For example, the cost per quitter for telephone counselling (in 2009–2010 prices) is £895, for minimal counselling plus NRT is £955, and for intensive counselling plus NRT is £1,621 [36]. The Flack et al. [5] study, which used the same model adopted in the present analysis, shows that among a range of interventions (combinations of self-help materials, nicotine patches, individual counselling and nicotine replacement therapy) the only intervention that was cost-increasing compared to brief GP advice was brief advice plus self help material plus nicotine replacement therapy (£1,074 per QALY, 2009–2010 prices). A subsequent study, using the same methodological assumptions, that evaluated the cost-effectiveness of a mass media campaign to prevent the uptake of smoking among young people, found using a range of sensitivity analyses compared to no intervention that the cost per QALY was never more than £1,142 per QALY (2009–2010 prices) [6].

The intervention is highly cost effective given the cost-effectiveness thresholds generally applied in the UK, and on the basis of these results the txt2stop mobile phone text messaging intervention should be considered as an addition to existing smoking cessation services. The self-reported smoking cessation results reported in the txt2stop trial are similar to those reported in previous trials of smoking cessation support delivered via mobile phone text messaging in New Zealand [3]. It would be technically relatively easy to scale up the intervention for delivery internationally, and it is likely that the intervention would be cost effective, at least in other high income settings that are likely to have similarly high cost savings as a result of reductions in smoking-related diseases.

Further research should establish the effects and cost-effectiveness of text-based smoking cessation support in other settings, such as low- and middle-income countries. Research is needed to evaluate the cost-effectiveness of text-based support, including the effects of smoking cessation on short-term health outcomes, and for long term smokers versus newer smokers.

This study clearly indicates that text-based support is likely to be a cost-effective means of encouraging smoking cessation and should be considered for inclusion in smoking cessation services.

## Appendix

See Tables 3, 4, 5, 6.

**Table 3:** Mortality rate per 1,000 by age and smoking status [15]

Age	Current smoker	Former smoker	Non-smoker
35–44	2.8	2	1.6
45–54	8.1	4.9	4
55–64	20.3	13.4	9.5
65–74	47	31.6	23.7
75–84	106	77.3	67.4
85+	218.7	179.7	168.6

**Table 4:** Relative risks (RRs) of chronic obstructive pulmonary disease (COPD), stroke, myocardial infarction (MI), lung cancer and coronary heart disease (CHD) by gender and smoking status [7]

	Current smoker	Former smoker	Non smoker
COPD			
Women	1	0.84	0.68
Men	1	0.96	0.92
Stroke			
Women/men	1.37	1.11	1
MI			
Women	1.6	1.11	1
Men	2.76	1.05	1
Lung cancer			
Women	1	0.44	0.03
Men	1	0.21	0.05
CHD			
Women/men	3.12	1.55	1

**Table 5:** Six-month probability of CHD, lung cancer, MI, stroke and COPD by age, gender and smoking status [7]

Age	Current smokers	Former smoker	Current smokers	Former smoker
	Female	Female	Male	Male
CHD				
16–24	0.0000	0.0000	0.0000	0.0000
25–34	0.0000	0.0000	0.0000	0.0000
35–44	0.0091	0.0045	0.0083	0.0041
45–54	0.0338	0.0166	0.0335	0.0165
55–64	0.1213	0.0583	0.1075	0.0519
65–74	0.2654	0.1218	0.2456	0.1135
75+	0.3674	0.1622	0.3234	0.1452
MI				
16–24	0.0000	0.0000	0.0000	0.0000
25–34	0.0000	0.0000	0.0000	0.0000
35–44	0.0000	0.0000	0.0000	0.0000
45–54	0.0000	0.0000	0.0000	0.0000
55–64	0.0730	0.0287	0.0465	0.0320
65–74	0.0772	0.0303	0.0485	0.0334
75+	0.1562	0.0597	0.0903	0.0617
COPD				
16–24	0.0045	0.0053	0.0065	0.0055
25–34	0.0044	0.0053	0.0062	0.0052
35–44	0.0045	0.0053	0.0063	0.0053
45–54	0.0044	0.0053	0.0063	0.0053
55–64	0.0045	0.0053	0.0061	0.0052
65–74	0.0225	0.0269	0.0315	0.0264
75+	0.0457	0.0546	0.0647	0.0541
Lung cancer				
16–24	0.0000	0.0000	0.0000	0.0000
25–34	0.0000	0.0000	0.0000	0.0000
35–44	0.0000	0.0000	0.0000	0.0000
45–54	0.0021	0.0009	0.0020	0.0009
55–64	0.0024	0.0011	0.0018	0.0008
65–74	0.0138	0.0061	0.0108	0.0047
75+	0.0155	0.0068	0.0115	0.0050
Stroke				
16–24	0.0000	0.0000	0.0000	0.0000
25–34	0.0000	0.0000	0.0000	0.0000
35–44	0.0020	0.0014	0.0020	0.0014
45–54	0.0082	0.0057	0.0082	0.0057
55–64	0.0143	0.0099	0.0143	0.0099
65–74	0.0552	0.0380	0.0552	0.0380
75+	0.0997	0.0680	0.0997	0.0680

**Table 6:** Parameter distributions used in the probabilistic sensitivity analysis (PSA)

Parameter		Distribution	
Probability of quitting at 6 months in the control group	0.049(SE: 0.4)	Beta	α = 1.28, *Β* = 26.75
RR of quitting at 6 month with T2S	2.20 (95 % CI 1.8–2.68)	Lognormal	Ln (SD) = −1.49, Ln (mean) = 0.98
Relapse rate after 1 year	0.30 (SE: 0.13)	Beta	α = 3.43, *Β* = 8
Health state values	Smoker 0.76 (SD: 0.12) Former smoker 0.78 (SD: 0.12) Stroke 0.58 (SD: 0.12) CHD 0.48 (SD: 0.12) Lung cancer: 0.80 (SD: 0.12) MI: 0.80 (SD: 0.12) COPD: 0.73 (SD: 0.12)	Beta	Smoker α = 153.27, *Β* = 48.40 Former smoker α = 82.88, *Β* = 23.38 Stroke α = 156.41, *Β* = 113.26 Lung cancer α = 141.42, *Β* = 35.36 MI α = 141.42, *Β* = 35.36 COPD α = 159.14, *Β* = 58.86 CHD α = 7.84, *Β* = 8.49
Baseline quit rate	0.01 (SE: 0.004)	Beta	α = 6.18, *Β* = 611.57
Unit cost of text-based intervention	£17.80 (SE: 25.49)	Gamma	α = 0.49, *Β* = 36.50
Unit cost of smoking-related disease	Lung cancer: £5,921 Stroke: £2,218 CHD: £1,144 MI: £2,341 COPD: £997	Gamma	Lung cancer α = 8.82, *Β* = 623.52 Stroke α = 1.24, *Β* = 166.19 CHD α = 0.33, *Β* = 3.226 MI α = 1.38, *Β* = 3.704 COPD α = 0.25, *Β* = 3.704
